# A Novel Bioanalytical Method for Determination of Inotodiol Isolated from Inonotus Obliquus and Its Application to Pharmacokinetic Study

**DOI:** 10.3390/plants10081631

**Published:** 2021-08-09

**Authors:** Jin Hyeok Kim, Dan Gao, Chong Woon Cho, Inkyu Hwang, Hyung Min Kim, Jong Seong Kang

**Affiliations:** College of Pharmacy, Chungnam National University, Daejeon 34134, Korea; oojh52@cnu.ac.kr (J.H.K.); gaodan521361@hotmail.com (D.G.); chongw113@naver.com (C.W.C.); hwanginkyu@cnu.ac.kr (I.H.)

**Keywords:** *Inonotus obliquus*, inotodiol, noncompartment analysis, pharmacokinetic study

## Abstract

In this study, we developed a bioanalytical method using liquid chromatography coupled to triple quadrupole tandem mass spectrometry (LC-MS/MS) to apply to a pharmacokinetic study of inotodiol, which is known for its anti-cancer activity. Plasma samples were prepared with alkaline hydrolysis, liquid–liquid extraction, and solid-phase extraction. Inotodiol was detected in positive mode with atmospheric pressure chemical ionization by multiple-reaction monitoring mode using LC-MS/MS. The developed method was validated with linearity, accuracy, and precision. Accuracy ranged from 97.8% to 111.9%, and the coefficient of variation for precision was 1.8% to 4.4%. The developed method was applied for pharmacokinetic study, and the mean pharmacokinetic parameters administration were calculated as follows: λ_z_ 0.016 min^−1^; T_1/2_ 49.35 min; C_max_ 2582 ng/mL; Cl 0.004 ng/min; AUC_0–t_ 109,500 ng×min/mL; MRT_0–t_ 32.30 min; Vd 0.281 mL after intravenous administration at dose of 2 mg/kg and λ_z_ 0.005 min^−1^; T_1/2_ 138.6 min; T_max_ 40 min; C_max_ 49.56 ng/mL; AUC_0–t_ 6176 ng×in/mL; MRT_0–t_ 103.7 min after oral administration. The absolute oral bioavailability of inotodiol was 0.45%, similar to nonpolar phytosterols. Collectively, this is the first bioanalytical method and pharmacokinetic study for inotodiol.

## 1. Introduction

Belonging to the family *Hymenochaetaceae*, *Inonotus obliquus*, known as chaga mushroom, is a polypore fungus collected from birch trees that have been used to treat cancer in Russia [1]. Many bioactive components have been isolated, and their pharmacological and biological studies have been also reported. For example, lanosterol, inotodiol, 3β-hydroxylanosta-8,24-dien-21-al, 3β,22R-dihydroxylanosta-8,24-dien-11-one, and ergosterol peroxide act on various cancer cells such as those of the Michigan cancer foundation-7 breast adenocarcinoma cell line [2] and the P388 and L1210 leukemia cell lines [3,4], and other studies also have reported antitumor activity from various component inhibiting the growth of COLO 205 gastric adenocarcinoma cells, HeLa cervical adenocarcinoma cells, A-549 lung carcinoma cells, and PC3 prostate carcinoma cells [5]. Among those bioactive components, inotodiol has been studied for various types of biological or pharmacological effects: not only just antitumor activity but also stabilizing mast cells to alleviate food allergy and inhibiting apoptosis of PC12 cells induced by oxygen/glucose deprivation/reoxygenation, which is effective in reversing the effects of ischemic stroke [6].

Previous studies have already demonstrated various bioactivities of inotodiol, but the mechanism has not been well elucidated. This is a common problem in the development of nutraceuticals or functional food [7]. Therefore, a bioanalytical study for a pharmacokinetic and pharmacodynamics study is necessary to better understand the pharmacological mechanism. There are few previous studies that developed analytical methods for inotodiol determination [8,9]. However, these methods were developed for the detection of inotodiol in the chaga mushroom, which is not suitable for the analysis of inotodiol in the biological matrix because the content and criteria of bioanalytical method validation are distinct from common analytical method validation. Therefore, developing a determination method of inotodiol in the biological matrix is needed.

To develop an analytical method for inotodiol determination, applying an analytical method for sterols should be considered since the structure of inotodiol is similar to sterols (Figure S1). Analysis of sterols, especially sterol lipids, is challenging because their solubilities are poor in blood and lipoproteins, phospholipids and triglycerides interrupt to analyze sterols. Moreover, the majority of sterols circulate as steryl esters, which are esterified with fatty acids, and a small portion of sterols circulate as free sterol form in animals [10]. These two forms of sterols make it more difficult to quantify in an animal sample.

The Bligh and Dyer method and the Folch method have been used to extract sterols [11,12]. In both methods, alkaline hydrolysis is applied to degrade abundant lipids, such as triglycerides and phospholipids, and to change esterified sterol into free sterol. Then, solid-phase extraction (SPE) is applied for the sample cleanup step to isolate sterols from the biological matrix. Although the extraction method for the analysis of sterols is well studied, a method for the analysis of inotodiol is lacking.

In this study, we developed a bioanalytical method to quantify inotodiol in plasma using high-performance liquid chromatography with a triple quadrupole mass spectrometer. Validation was performed to evaluate the validity of the method, and a pharmacokinetic study was conducted to confirm the applicability. This is the first bioanalytical and pharmacokinetic study of inotodiol, and this study can be applied for a better understanding of absorption, distribution, metabolism, excretion, and toxicity.

## 2. Results and Discussion

### 2.1. Sample Preparation

Sample preparation steps consist of alkaline hydrolysis, liquid-liquid extraction and SPE. Suitable temperature and time are essential for alkaline hydrolysis since high temperature can cause degradation of sterol compounds as well as inotodiol. Samples prepared with alkaline hydrolysis showed more precise results with regard to the coefficient of variation (Table 1). The alkaline hydrolysis step significantly improved the coefficient of variation, which made the method suitable in accordance with the guideline on bioanalytical method validation of the European Medicines Agency (EMA), which recommends maintaining lower than 5% for the coefficient of variation in precision. Therefore, the alkaline hydrolysis step was included in the sample preparation steps.

After hydrolysis, dichloromethane (DCM) was used in this study to extract inotodiol. Hexane, DCM, and chloroform were commonly used for the extraction of sterols and oxysterols because of their low polarity. Chloroform and n-hexane are more toxic than DCM. In addition, phosgene can be formed in chloroform, which could react with KOH to form dichlorocarbene [13].

### 2.2. Instrumental Analysis

Acetonitrile (ACN) and methanol with water are commonly used as a mobile phase with water for reverse-phase chromatography [14]. ACN was selected as a mobile phase in this study because it has stronger elution strength than methanol. Inotodiol has low polarity and should be analyzed with ACN to reduce the elution time.

Liquid chromatography coupled to triple quadrupole tandem mass spectrometry (LC–MS/MS) has been widely used to develop bioanalytical methods since it has good sensitivity and selectivity toward a complex sample matrix. As mass spectrometry is able to analyze the ionized compounds, the ionization method of inotodiol should be considered to acquire optimized results. Sterols are nonpolar compound which is not susceptible to ionization with electrospray ionization. Since the structure of inotodiol resembles sterols, ionization with atmospheric-pressure chemical ionization (APCI) interface was applied with neutral mobile phase without acidic additives [13,15]. 

**Figure 1:**
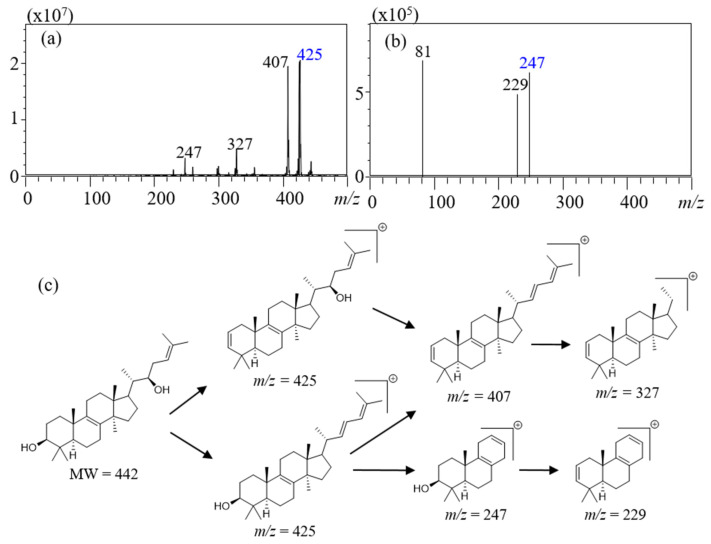
Atmospheric pressure chemical ionization coupled to tandem mass spectrometry and proposed fragmentation pathway; (**a**) precursor ion spectrum, (**b**) product ion spectrum, and (**c**) proposed fragmentation pathway. Mass spectrometry conditions: Interface; APCI, nebulizing gas flow; 3 L/min, interface temperature; 350 °C, desolvation line temperature; 200 °C, heat block temperature; 200 °C, drying gas flow; 5 L/min.

Inotodiol showed high intensity at positive modes because it has two hydroxyl groups that normally are positively charged. The most common ionization form of hydroxyl groups is the protonated form [M+H]^+^, and the second most common form is the loss of a hydroxyl group [M-OH]^+^ [16]. Inotodiol, which has a molecular weight of 442, is ionized as the later form, and so the mass to charge ratio (*m*/*z*) value of the selected precursor ion was 425 at positive mode Figure 1a. The internal standard, triamcinolone acetonide (Figure S2), was detected in [M+H]^+^ with an *m*/*z* value of 435. The selected-reaction monitoring program was set to monitor precursor-to-product-ion transitions for inotodiol (*m*/*z* 425→247) and triamcinolone (*m*/*z* 435→339) at −15 eV of the collision energy. According to the literature about the fragmentation pathway of sterols [17], the fragmentation process is illustrated in Figure 1c with MS/MS spectra. The precursor ion showed *m*/*z* 425, which means that carbocation occurred after dehydration [M-H_2_O+H]^+^. Inotodiol has two hydroxyl groups, which can be lost. Indeed, the signal observed at *m*/*z* 407 with similar intensity as one at *m*/*z* 425 corresponds to the removal of two hydrogen groups, [M-2H_2_O+H]^+^. A double bond after dehydration subsequently led to the loss of the side chain, which produced *m*/*z* 327 by a retro-ene reaction. In the second MS spectra (Figure 1b), signals with similar intensities were observed at *m*/*z* 247 and *m*/*z* 229. The mass difference between two ions (−18) indicated dehydration. Therefore, the fragment *m*/*z* 247 containing one hydroxyl group could be formed by ring cleavage of cyclopentane, which would lead to ring opening, loss of neutral ethylene, and the formation of a double bond [18].

### 2.3. Bioanalytical Method Validation

**Figure 2:**
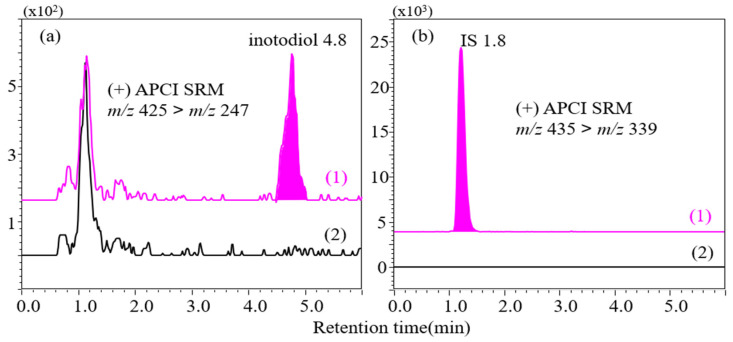
Selected reaction monitoring chromatograms of (**a**) inotodiol and (**b**) triamcinolone acetonide used as internal standard (IS); (**a**1) 4 ng/mL inotodiol in plasma and (**a**2) blank plasma; (**b**1) 150 ng/mL IS in plasma and (**b**2) blank plasma. Liquid chromatographic conditions: column; HECTOR-M C8 (75 × 2.1 mm, 3 µm), flow rate; 0.2 mL/min, eluent; 85% acetonitrile (isocratic).

Specificity was confirmed by analysis of blank plasma and the lower limit of quantification (LLOQ) sample. The peaks of inotodiol and the internal standard were not detected with the developed the LC–MS/MS method in blank plasma samples from seven different mice (Figure 2).

The formula of calibration curve was y = 0.0053x − 0.0015 (*n* = 6). The calibration curve of inotodiol showed good linearity in the range of 4–300 ng/mL; the coefficient of determination was 0.9993. Additionally, the back-calculated concentrations of calibration standards were within 15% except that of LLOQ; the back-calculated concentration of LLOQ was less than 20% (Table S1).

The inotodiol peak was not observed in the blank sample (Figure S3) after analysis of the upper limit of the quantification (ULOQ) sample. This result confirmed that the previous run did not affect the later run for detecting inotodiol, and carryover was not observed.

Precision and accuracy were suitable for LLOQ and quality control samples (Table 2). The coefficient of variation for within-run precision ranged from 1.8% to 3.5%, and for between-run precision ranged from 3.6% to 4.4%, which accepted range was within 5%. Within-run accuracy ranged from 97.8% to 111.9%, and between-run accuracy ranged from 104.8% to 112.2%. The accepted range was from 85% to 115% except for the LLOQ sample.

The internal standard–normalized matrix factors and their coefficients of variation for quality control samples are listed in Table S2. These matrix factors and low value of coefficients of variation indicate that the developed method reduced matrix effects, and the matrix did not significantly affect the analysis of inotodiol.

Table 3 showed the analysis results for stability of quality control samples. The ratios of the calculated concentration to the nominal concentration ranged from 96.2% to 99.8% for freeze-thaw stability and from 101.8% to 108.7% for short-term stability. Lastly, the percentages of calculated concentration divided by nominal concentration ranged from 100.2% to 112.5% for long-term stability.

The coefficient of variation for diluted samples ranged from 1.8% to 3.7%, and accuracy ranged from 94.8% to 107.5% (Table S3). Therefore, samples with concentrations higher than the ULOQ can be quantified by dilution.

### 2.4. Application of Pharmacokinetic Study

**Figure 3:**
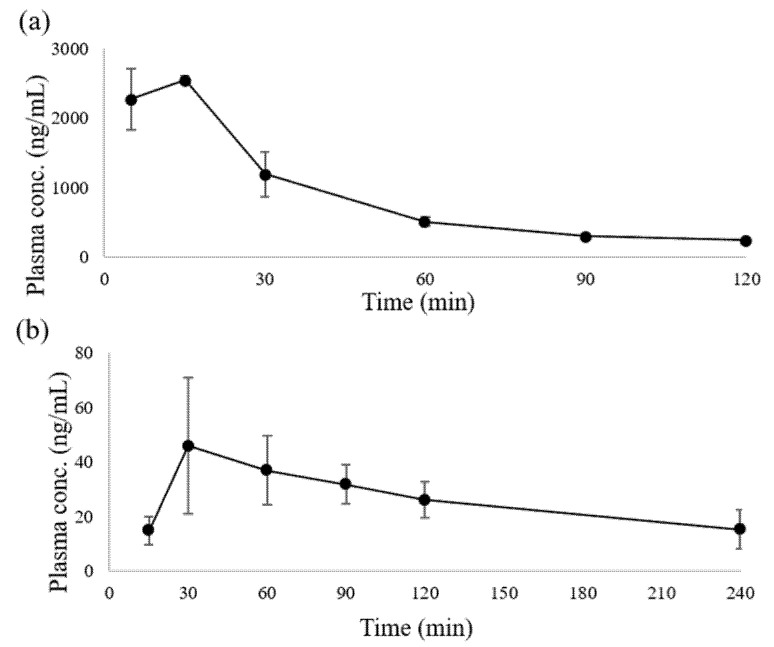
The mean plasma inotodiol concentration–time curve after (**a**) intravenous bolus administration at a dose of 2 mg/kg and (**b**) oral bolus administration at a dose of 20 mg/kg.

The developed method was applied to quantify inotodiol in plasma after oral and intravenous bolus administration. The mean plasma concentration-time curve is illustrated in Figure 3. In total, nine pharmacokinetic parameters were estimated: first-order rate constant associated with terminal portion of the log-linear curve (λ_z_), half-life (T_1/2_), peak time (T_max_), peak plasma concentration (C_max_), clearance (Cl), area under the plasma drug concentration-time curve from time zero to the latest measurable time (AUC_0–t_), mean residence time (MRT_0–t_), volume of distribution (Vd), and bioavailability (F, fraction of drug absorbed), and they are listed in Table 4.

After oral administration, inotodiol showed poor absorption with a mean C_max_ of 49.56 ng/mL and T_max_ of 40 min. AUC after oral administration was found to be 6176 ng×min/mL. After intravenous administration, Vd was estimated as 0.281 mL, which refers to poor distribution of inotodiol in organs and tissues. The absolute bioavailability is a very important parameter because bioavailability indicates delivery to the systemic circulation. The mean F was 0.45%, and such low F means that inotodiol was almost wasted. This poor F is known to result from poor intestinal absorption. According to a previous study, intestinal absorption of beta-sitosterol and campesterol were 0.42% and 0.63% after oral and intravenous administration, which were similar to our data [19]. 

Further study to improve absorption of inotodiol, such as investigation of pharmaceutical dosages and routes of administration, seems necessary to determine functional foods or health supplements. Additionally, a pharmacokinetic study with compartmental analysis should be conducted to better understand absorption, distribution, metabolism, and excretion of inotodiol.

### 2.5. Computational Prediction of Pharmacokinetics

**Figure 4:**
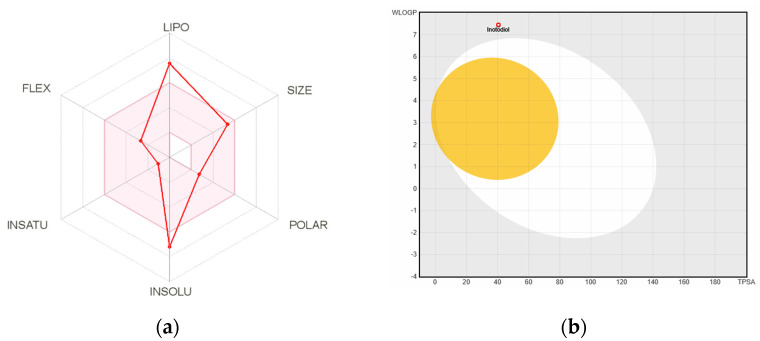
Computational prediction of bioavailability, gastrointestinal absorption, and brain penetration by SwissADME. (**a**) The bioavailability radar; LIPO: lipophilicity; POLAR: polarity; INSOLU: solubility; INSATU: saturation; FLEX: flexibility. (**b**) Boiled egg model for intuitive evaluation of passive gastrointestinal absorption and brain penetration.

The bioavailability radar (Figure 4a) provides information on the drug-likeness of a molecule, which describes lipophilicity, size, polarity, solubility, and saturation flexibility [20]. A pin area in the radar represents a suitable physicochemical range for oral bioavailability. According to the radar, inotodiol is not orally available because it is too lipophilic and insoluble. This finding corresponds to the absolute bioavailability described in Section 2.4.

Lipophilicity was presented by the partition coefficient between n-octanol and water (log Po/w), which is calculated by several computational methods. The log Po/w was 6.62, which indicates that inotodiol is poorly soluble in water.

The white region of the boiled egg model (Figure 4b) represents a high probability of passive absorption by the gastrointestinal tract, and the yellow region (yolk) represents a high probability of brain penetration. Since inotodiol is positioned in the outer area of the boiled egg region, we could speculate that inotodiol has poor gastrointestinal absorption and rarely permeate the blood–brain barrier. 

## 3. Materials and Methods

### 3.1. Materials

Potassium hydroxide and polysorbate 80 (Tween 80) were purchased from Samchun Chemicals (Pyeongtaek, Republic of Korea). Triamcinolone acetonide, purchased from Acros Organics (Incheon, Republic of Korea), was used as the internal standard. Guaranteed-grade reagents such as n-hexane, chloroform, acetone, methyl alcohol, and ethyl alcohol for isolation were purchased from SK Chemicals (Seongnam, Republic of Korea). Water was purified using the Milli-Q system by MilliporeSigma (Burlington, MA, USA). High-performance liquid chromatography-grade ACN and DCM were purchased from Honeywell–Burdick & Jackson (Muskegon, MI, USA) for instrumental analysis. 

### 3.2. Animals

Eight-week-old female BALB/c mice were purchased from Samtako BioKorea Co. (Osan, Republic of Korea) and accommodated in the Core Animal Facility in Chungnam National University, Daejeon, Republic of Korea. The animal study protocol used in this study was approbated by the Animal Ethics Committee of Chungnam National University (Approval Number: CNU-00570), and animal experiments followed the approbated protocol. The animals were housed for 1 week before the experiment.

### 3.3. Isolation and Purification of Inotodiol

The spectra (hydrogen-1 and carbon-13) were obtained on a 400-MHz Fourier transform nuclear magnetic resonance spectrometer (Bruker, Billerica, MA, USA), the standard for which was tetramethylsilane. Medium-pressure liquid chromatography was used on an Isolera One system (Biotage, Uppsala, Sweden) and silica gel mesh (70–230 mesh and 230–400 mesh; Merck, Whitehouse Station, NJ, USA) and YMC reversed-phase (RP)–18 resins (75 µm; Fuji Silysia Chemical Ltd., Kasugai, Japan) were used as absorbents in the column chromatography. Thin-layer chromatography with the YMC RP-18 resins was performed with the use of precoated silica gel 60 F254 and RP-18 F254S plates (both 0.25 mm; Merck, Darmstadt, Germany), and the spots were detected under ultraviolet light at 254 and 365 nm wavelengths and with 10% H2SO4, followed by heating for 3 to 5 min.

Pulverized *I. obliquus* (2.0 kg) was freeze-dried and extracted with 6 L ethanol in ultrasonic conditions for 6 h. Then, ethyl alcohol residue (600 g) was obtained after the evaporation of the solvent under reduced pressure. This residue was suspended in water and partitioned with DCM to produce DCM residue (200 g) and a water layer, respectively. The DCM residue was separated by elution with silica gel column chromatography with combinations of n-hexane and acetone (100:1, 50:1, 25:1, 10:1, and 5:1) to produce five subfractions (C-1 to C-5). Inotodiol (400 mg) was separated from subfraction C-2 (10.0 g) by medium-pressure liquid chromatography using acetone and water (3:1, *v*/*v*) as mobile phase.

Inotodiol: White crystals, C_30_H_50_O_2_, 1H-NMR (δH ppm, 400 MHz); (0.67, 3H, H-18), (0.93, 3H, H-19), (0.88, 3H, H-21), (1.60, 3H, H-26), (1.69, 3H, H-27), (0.90, 3H, H-28), (0.76, 3H, H-29), and (0.82, 3H, H-30); 13C-NMR δC ppm (100 MHz): 35.4 (C-1), 27.6 (C-2), 78.7 (C-3), 38.7 (C-4), 50.2 (C-5), 18.9 (C-6), 26.3 (C-7), 134.4 (C-8), 134.0 (C-9), 36.8 (C-10), 20.8 (C-11), 28.9 (C-12), 44.5 (C-13), 49.2 (C-14), 30.7 (C-15), 30.7 (C-16), 47.0 (C-17), 15.5 (C-18), 18.0 (C-19), 41.5 (C-20), 12.4 (C-21), 73.2 (C-22), 27.0 (C-23), 121.2 (C-24), 134.8 (C-25), 25.7 (C-26), 17.9 (C-7), 27.7 (C-28), 15.2 (C-29), and 24.1 (C-30).

### 3.4. Sample Preparation

A stock solution of inotodiol was prepared at a concentration of 0.25 mg/mL of ACN. Working solutions were prepared by diluting the stock solution with ACN. The final concentrations of working solutions were 900, 690, 600, 360, 300, 150, 120, 75, and 12 ng/mL for calibration curve and quality control samples. A stock solution of the internal standard was prepared at a concentration of 0.65 mg/mL in ACN and then diluted to 450 ng/mL with ACN.

A total of 30 μL of the working solutions of inotodiol was added to 20 μL of plasma in 2-mL Axygen microcentrifuge tubes (Corning Incorporated, Corning, NY, USA). Then, 300 μL of 6 M KOH and methanol were also added. The tube was incubated at 55 °C for 90 min in a JSOF-Series forced convection oven (JS Research, Inc., Tokyo, Japan) to achieve alkaline hydrolysis. After incubation, 1 mL of DCM was added to the tube, and the sample was centrifuged at 325,953× *g* relative centrifugal field (RCF) for 6 min in a Smart R17 centrifuge (Hanil Scientific, Daejeon, Republic of Korea). The supernatant was decanted into another tube, and the residual DCM layer was evaporated by nitrogen gas. Then, 1 mL of DCM was added to the tube containing supernatant, and the tube was centrifuged as the earlier method. The upper layer was removed, and the lower layer was decanted into the tube. Lastly, the transferred solvent was evaporated by nitrogen gas, and 0.2 mL of 70% ACN was added to dissolve the extracts.

The next step was SPE. Inotodiol was extracted by Sep-Pak C18 3 cc Vac silica SPE column (Waters Corporation, Milford, MA, USA). The column was conditioned with 3 mL of ACN. The sample was loaded into the SPE column, and then the column was flushed with 5 mL of 70% ACN and 0.4 mL of ACN. Finally, inotodiol was eluted by 7 mL of ACN, and the eluent was placed in a 10-mL glass test tube. The sample was dried completely with nitrogen at 45 °C. Lastly, 60 μL of ACN and 30 μL of the internal standard working solution were added to the test tube, and it was sonicated for 30 s. The sample was filtered through a 0.2 μm polyvinylidene fluoride (PVDF) syringe filter (Whatman plc, Maidstone, UK) for instrumental analysis.

### 3.5. Instrumental Conditions

To perform liquid chromatography coupled to triple quadrupole tandem mass spectrometry (LC-MS/MS) analysis, a Prominence UFLC system (Shimadzu, Kyoto, Japan) was connected to an LCMS-8040 system (Shimadzu). The Prominence UFLC system was equipped with a degasser, a column oven, a pump, a module and an autosampler. After a 10 μL injection of the sample, analytes were identified through a HECTOR-M C8 high-performance liquid chromatography column (75 × 2.1 mm, 3 μm; RStech Corporation, Daejeon, Republic of Korea). The elution started from an 85 % ACN for 6 min, increased to 100% ACN immediately, held for 11 min, returned to 85 % ACN and equilibrated for 6 min at a flow rate of 0.2 mL/min. Detection was carried out by the APCI by multiple-reaction monitoring mode. Inotodiol and the internal standard were ionized mainly as [M-OH]^+^ and [M+H]^+^. Inotodiol was fragmented to *m*/*z* values of 229 and 247, and the internal standard was transmitted at *m*/*z* values of 339, 397, and 415.

### 3.6. Bioanalytical Method Validation

The developed method was validated in accordance with the guideline by EMA for selectivity, carryover, LLOQ, calibration curve, accuracy, precision, matrix effect, and stability [21]. Blank plasma for validation was obtained from seven different mice and was used for selectivity. Selectivity was proven by analysis of the blank plasma and spiked plasma samples. Carryover was evaluated in blank samples after analysis of the ULOQ sample. LLOQ was calculated from the signal-to-noise ratio. To draw the calibration curve in the range of 4 to 300 ng/mL, the blank matrix was spiked with six concentrations. The back-calculation was the calculated concentration by calibration divided by nominal concentration to prove the suitability of calibration.

To validate within-run precision and accuracy, quality control samples and LLOQ samples were analyzed six times. To determine between-run precision and accuracy, quality control and LLOQ samples were analyzed in different batches for 3 consecutive days. All samples for analysis of precision and accuracy were freshly made. Precision was calculated as the coefficient of variation, and accuracy was calculated as percentages of the nominal value divided by the value calculated from the calibration curve. The matrix effect (also known as recovery) was determined by the internal standard-normalized matrix factor calculated as the ratio of peak area in the presence of matrix to the peak area in the absence of matrix. To assess stability, quality control samples were spiked with three different conditions in triplicates for short-term stability (2 days at room temperature), long-term stability (30 days at −80 °C) and freeze-and-thaw stability (two freeze/thaw cycles at −80 °C). The dilution integrity was assessed by mean accuracy, and to obtain the coefficient of variation, five replicates of one-third of the sample that was diluted (from 330, 500, and 700 ng/mL) were analyzed to quantify samples with concentrations higher than the ULOQ.

### 3.7. Pharmacokinetic Study

Inotodiol was suspended in 0.5% polysorbate 80 and administered to two groups of mice for the pharmacokinetic study. In one group, a dose of 2 mg/kg was administered by intravenous bolus injection, and blood samples were collected in tubes containing heparin 5, 15, 30 60, 90, 120 min after intravenous administration. In the other group, a dose of 20 mg/kg was orally administered as a bolus, and blood samples were collected into heparinized tubes 15, 30, 60, 90, 120, 240 min after oral administration. Obtained blood samples were centrifuged at 1124205 RCF for 5 min to isolate plasma, and all samples were stored at −80 °C before sample preparation. The noncompartment package of RStudio (freeware version 3.5.1) was used to estimate pharmacokinetic parameters.

The C_max_ and T_max_ were obtained from experimental data. λ_z_ was calculated as a slope of the linear regression, which was drawn as a semi-log plot. The T_1/2_ was calculated by 0.693/λ_z_. The AUC_0–t_ was calculated from the plasma drug concentration-time curve in accordance with the trapezoidal rule. Cl was calculated as λ_z_ × Vd. MRT_0–t_ was calculated as AUMC_0–t_/AUC_0–t_, which AUMC_0–t_ means the area under the first moment curve. F was calculated as the dose-corrected AUC after oral administration divided by AUC after intravenous administration.

### 3.8. Computational Pharmacokinetic Study

Pharmacokinetic properties were predicted using SwissADME (http://www.swissadme.ch/; retrived 7 November 2020) to compare the results of this pharmacokinetic study with the computational result and to predict additional physicochemical properties, absorption, distribution, metabolism, elimination, and toxicity properties. The isomeric simplified molecular input line entry system was found on Pubchem (https://pubchem.ncbi.nlm.nih.gov/; retrived 7 November 2020) and was used as input data for the computational study.

The bioavailability radar included six axes with six major properties for oral bioavailability. The pink area illustrated optimal values of six properties. Six parameters were estimated as follows: Saturation, the ratio of sp3 hybridized carbons over the total carbon count of the molecule should be at least 0.25; Size, the molecular weight calculated should be between 150 and 500 g/mol for size; Polarity, the topological polar surface area should be between 20 and 130 Å^2^. Solubility, log S calculated with the estimating aqueous solubility directly from molecular structure mode should not exceed 6; Lipophilicity, octanol/water partition coefficients by guiding an additive model should be in the range from −0.7 to +6.0; Flexibility, the molecule should not have more than 9 rotatable bonds.

Passive gastro-intestinal absorption and blood-brain barrier permeation are predicted with the BOILED-Egg model, which defines favorable and unfavorable zones in the log Po/w versus polar surface area physicochemical space for passive diffusion through both physiological barriers.

## 4. Conclusions

To date, many studies have evaluated the bioactivities of inotodiol. However, pharmacokinetic and bioanalytical studies have not been reported. In this study, a reliable LC–MS/MS method focusing on precise and accurate quantification of inotodiol in mouse plasma was developed. This method was applied to pharmacokinetic profiles after intravenous and oral bolus administration of 2 mg/kg and 20 mg/kg of inotodiol, respectively. The developed method was validated in accordance with the guidelines of the EMA. Inotodiol was successfully detected in plasma, and pharmacokinetic profiles were well defined. The pharmacokinetic results revealed poor bioavailability. Therefore, oral administration of inotodiol is not an efficient way of administration. As such, other routes of administration or formulation should be further studied. Additionally, computational prediction results supported and explained the pharmacokinetic results. This is the first bioanalytical and pharmacokinetic study of inotodiol and can be employed in assessing metabolic profiles and other investigations.

## Figures and Tables

**Table 1:** Plasma concentration of inotodiol (ng/mL) at 30 min after oral bolus administration with or without alkaline hydrolysis step.

	Dose of 40 mg/kg		Dose of 20 mg/kg	
	Without Hydrolysis	With Hydrolysis	Without Hydrolysis	With Hydrolysis
1	108	123	64	77
2	115	118	58	74
3	130	121	67	80
Mean	118	121	63	77
SD ^1^	9.0	2.0	4.0	2.0
CV ^2^	8.0	2.0	6.0	3.0

^1^ SD: standard deviation, ^2^ CV: coefficient of variation (%).

**Table 2:** Within-run and between-run precision and accuracy of inotodiol (*n* = 3).

**Within Run**			
**Conc (ng/mL) ^1^**	**Determined (ng/mL)**	**Accuracy (%)**	**CV (%) ^2^**
4	4.5 ± 0.1	111.9	1.8
10	9.8 ± 0.2	97.8	2.2
120	124.3 ± 4.4	103.6	3.5
230	233.0 ± 4.7	101.3	2.0
**Between Run**			
**Conc (ng/mL)**	**Determined (ng/mL)**	**Accuracy (%)**	**CV (%)**
4	4.5 ± 0.2	112.2	3.6
10	10.7 ± 0.4	107.3	3.6
120	125.7 ± 4.2	104.8	3.4
230	247.0 ± 11.0	107.5	4.4

^1^ Conc: concentration; ^2^ CV: coefficient of variation.

**Table 3:** The stability of inotodiol in quality control samples (*n* = 3).

**Freeze-Thaw Stability (Two Freeze/Thaw Cycles at −80 °C)**			
**^1^ Conc** **(ng/mL)**	**Calculated (ng/mL)**	**Accuracy** **(%)**	**^1^ CV** **(%)**
10	10.0 ± 0.4	99.8	3.6
120	115.5 ± 1.9	96.2	1.6
230	228.1 ± 4.3	99.2	1.9
**Short-Term Stability (Storage at Room Temperature for 2 Days)**			
**^1^ Conc** **(ng/mL)**	**Calculated (ng/mL)**	**Accuracy** **(%)**	**^2^ CV** **(%)**
10	10.2 ± 0.4	101.8	3.7
120	125.2 ± 2.0	104.3	1.7
230	250.0 ± 6.4	108.7	2.6
**Long-Term Stability (Storage for 30 Days at −80 °C)**			
**^1^ Conc** **(ng/mL)**	**Calculated (ng/mL)**	**Accuracy** **(%)**	**^2^ CV** **(%)**
10	10.0 ± 0.62	100.2	6.2
120	131.1 ± 3.0	109.3	2.3
230	258.8 ± 2.7	112.5	1.0

^1^ Conc: concentration; ^2^ CV: coefficient of variation.

**Table 4:** Pharmacokinetic parameters of inotodiol after intravenous bolus administration and oral bolus administration (*n* = 3 for each group).

Parameters	^1^ Intravenous	^1^ Oral
λ_z_ (1/min)	0.016 ± 0.01	0.005 ± 0.00
T_1/2_ (min)	49.35 ± 14.1	138.6 ± 30.3
T_max_ (min)		40 ± 14.1
C_max_ (ng/mL)	2582 ± 74.2	49.56 ± 14.2
Cl (ng/min)	0.004 ± 0.00	
AUC_0–t_ (ng×min/mL)	109500 ± 5670	6176 ± 455
MRT_0–t_ (min)	32.30 ± 0.55	103.7 ± 3.54
Vd (mL)	0.281 ± 0.08	
F (%)		0.45 ± 0.0

^1^ data was represented as mean ± standard deviation.

## Data Availability

Not applicable.

## Supplementary Materials

The following are available online at https://www.mdpi.com/article/10.3390/plants10081631/s1, Figure S1: Chemical structure of (a) inotodiol and (b) cholesterol, Figure S2: Chemical structure of Triamcinolone acetonide used for internal standard (IS), Figure S3: SRM of inotodiol for carry over (injection order: (1) ULOQ sample, (2) blank sample), Figure S4: The boiled egg model for intuitive evaluation of passive gastrointestinal absorption and brain penetration by Swiss ADME, Table S1: Percentages of the back-calculated concentration from the equation of calibration curve for inotodiol (*n* = 3), Table S2: IS-normalised matrix factor and coefficient of variation of quality control samples, Table S3: Dilution integrity of inotodiol after 3 times dilution (*n* = 5).
