# Supplementary material for: A Novel Bioanalytical Method for Determination of Inotodiol Isolated from Inonotus Obliquus and Its Application to Pharmacokinetic Study
Source: Plants (Basel). 2021 Aug 9;10(8):1631. doi: 10.3390/plants10081631 (PMC8401913; doi:10.3390/plants10081631)
Supplement: Supplementary file 1 [file plants-10-01631-s001.zip › plants-1292953-supplementary.pdf]

SUPPLEMENTARY MATERIAL

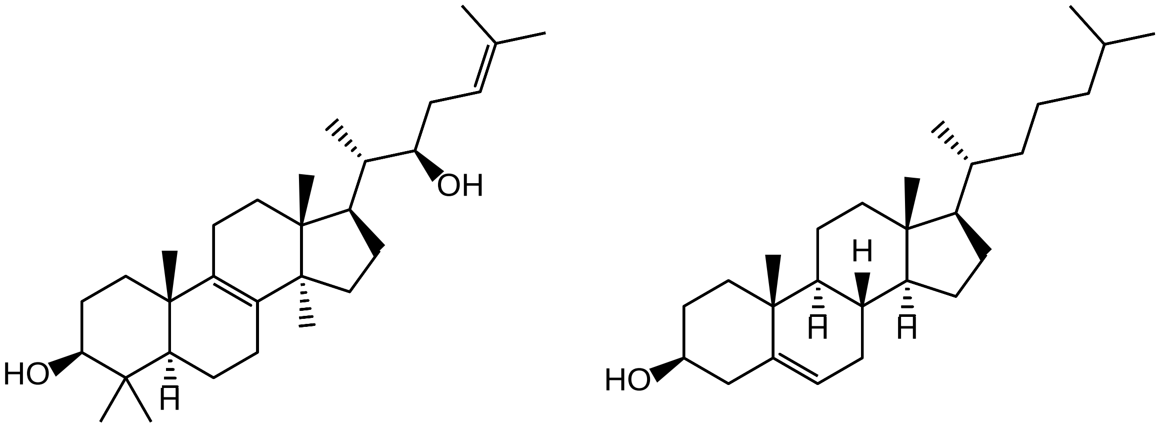

(a) (b)

Figure S1 Chemical structure of (a) inotodiol and (b) cholesterol.

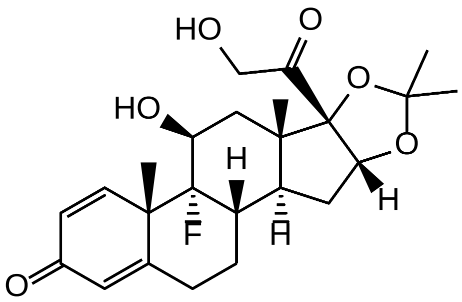

Figure S2 Chemical structure of Triamcinolone acetonide used for internal standard (IS).

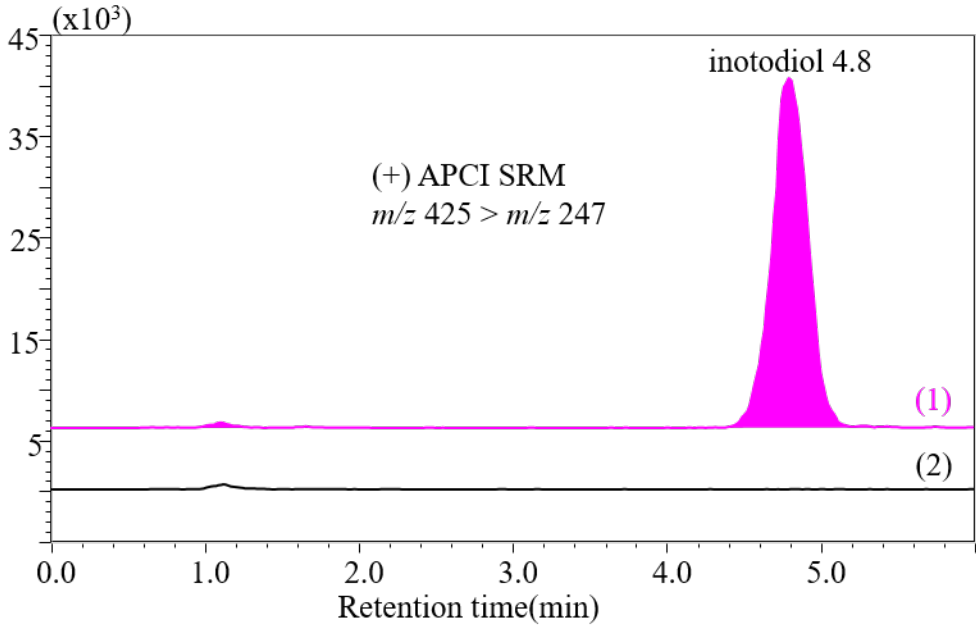

Figure S3 Selected reaction monitoring chromatogram of inotodiol for carry over (injection order: (1) ULOQ sample, (2) blank sample).

Table S1 Percentages of back calculated concentration from equation of calibration curve for inotodiol (n=3).

Amount (ng/mL)	Peak area of inotodiol	Peak area of IS	Area ratio of inotodiol/IS	Back calculation (%)
4	8510	357000	0.0238	117.1
	5760	268000	0.0215	106.1
	6890	303700	0.0227	111.7
25	46700	348600	0.1339	111.8
	42800	291500	0.1468	111.5
	47400	325300	0.1458	110.8
50	91000	378200	0.2407	91.2
	85100	322600	0.2637	99.9
	84200	318000	0.2647	100.3
100	177700	354100	0.5017	94.8
	160200	301600	0.5310	100.4
	190100	336000	0.5657	106.9
200	388100	398900	0.9729	91.9
	306800	297700	1.0303	97.3
	378500	349700	1.0821	102.2
300	527800	354700	1.4880	93.6
	518100	310700	1.6676	104.9
	507500	298600	1.6997	106.9

Table S2 Internal standard-normalised matrix factor and coefficient of variation of quality control samples.

	MF of low QC	MF of middle QC	MF of high QC
Sample 1	105.5	107.0	95.7
Sample 2	115.8	108.1	101.2
Sample 3	109.9	107.8	102.6
Sample 4	111.9	108.4	96.7
Sample 5	107.1	105.7	101.3
Mean	110.0	107.4	99.5
CV	3.3	0.9	2.8

Table S3 Dilution integrity of inotodiol after 3 times dilution (n=5).

Original concentration (ng/mL)	Found after dilution mean ± SD	Mean accuracy (%)	CV (%)
330	104.3 ± 3.8	94.8	3.6
500	171.9 ± 6.3	107.5	3.7
700	243.4 ± 4.3	104.5	1.8
